# The Essential Oil of Tobacco, a German Remedy for Tooth-Ache, with the Mode of Preparing It and the Three Cases Which Deterred the Writer from Ever Attempting to Administer It Again

**Published:** 1854-01

**Authors:** J. L. Levison


### ARTICLE VII.

*The Essential Oil of Tobacco, a German Remedy for Tooth-Ache, with the mode of preparing it and the three cases which deterred the writer from ever attempting to administer it again.* By J. L. LEVISON, D. D. S.

HAVING read some papers in your excellent periodical, on the effects of tobacco on the teeth, et cetera, it occurred to me that the following might be acceptable. Not so much for any scientific value of the communication, but as indicating the absurdity of any empirical treatment, particularly when the remedial agent is a powerful poison. Tobacco is known to be a most dangerous narcotic and stimulant, and when used without due care, death has often resulted, from even an infusion of the weed when injected *per ano;* but how much more potent and dangerous it is in its immediate effects on the brain and nervous system in general, and on the nerves of the stomach in particular, when administered in the form of the essential oil of this plant, in ordinary tooth-ache. The stomach being in such case immediately affected through the mucous surfaces, and the brain afterwards.

Prior to giving my own experience, it is essential to state my method of preparing the essential oil. I procured a clay-pipe, filled it with tobacco, and on the extremity of the tube a mouth-piece was put, cut from the lower part of a goose-quill, and into this tube or mouth-piece I inserted a portion of white cotton wool. The tobacco was then ignited, and a few streams of smoke were drawn through the cotton into the mouth, and from the latter sent in graceful curls into the circumambient air. A few seconds sufficed to saturate the cotton with a dark yellowish oil.

*Case* 1.—The writer's sister had a very carious tooth, but from a sense of fear would not have it extracted. As she suffered excruciating pain, which none of the usual palliatives relieved, she was persuaded to try the oil of tobacco, and it was prepared after the above method.

The cotton thus saturated with essential oil, was inserted in the hollow tooth, and in an instant the pain ceased, but the after consequences were indeed most frightful. The young lady was entirely prostrated, and was subsequently sick, and purged for nearly three days, like in the autumnal English cholera. In fact she was poisoned. But her consolation for this temporary inconvenience and punishment, was regarded by her as less annoying than her previous neuralgic suffering. Yet she would not have recourse to it again.

Case 2.—Being on a visit to a Unitarian minister, an habitual smoker, he said whilst we were walking together in his garden, "my dear sir, I wish you could cure the tooth-ache of Susan our cook, as she is suffering extreme agony; and has lost so many teeth, that she is reluctant to part with the few which remain." I answered, that I would try if he would furnish me with a pipe and the other materials required. When all was ready, Susan was summoned to appear in the paved court-yard before the house, which, with the garden, was enclosed by a high wall. It was a beautiful summer morning, and she came most willingly. After explaining the action of the remedy, I administered it, cautioning her to reject the cotton wool speedily, and on no account to swallow the saliva. The patent cotton was then put into the tooth, and she immediately lost all the pain. I then left her to resume my *tete a tete* with her learned master, but turned round to ascertain if Susan had gone to resume her vocation in the *cuisine*, when to my horror I saw her gyrating in such a rapid manner, like one in a state of extreme intoxication, that I ran back with the speed of a grey-hound, and just came in time to save her from falling, which would have been attended with some permanent consequence, as she was a very fat woman. So all the *fee* I earned, consisted in using my utmost effort to assist the servants to convey a senseless piece of humanity to her bed-room, where I labored for some time to restore her to consciousness. When this was effected, she was quite free from pain.

Case 3.—My last case was likely to be attended with some personal danger. A very tall and powerful farmer came into a shop in which I was at giving some orders, when he said, "I am mad with the tooth-ache." The shopman, who knew me, answered, "well this gentleman can cure you." "Ah!" sighed the yeoman, "but not without taking out the tooth, and I had my jaw nearly broken when a doctor tried it some time since." I proposed the German remedy, and retired into a back room to prepare it. There my stalwart patient came on being summoned. The cure was instantaneous, and I could have exclaimed with Cæsar, "*veni, vidi, vici.*" For with a regular halloo, he shouted, "the pain's gone! the pain's gone." And as I did this, *sans* a fee, I left immediately afterwards. Particularly as the robust young gentleman seemed quite well. Soon afterwards he was taken very sick and dizzy, was conveyed to the inn, and had to go to bed. When he recovered he sought for me every where, and threatened "to give me a sound drubbing;" so as this sort of practice was profitless, I have abandoned it ever since.

Yet with some greater precaution, the dental practitioner might have recourse to this remedy in bad cases, whenever the patient had a dread of extraction.

If I ever ventured to use it again, I would have an alkaline lotion, and after inserting the patent anti-neuralgic remedy, would give some of it to rinse the mouth, as the alkali would combine with the oil and form soap, and then probably the unpleasant effects might be prevented, which had hitherto been the result of its application. When my health is better, I hope to send you a paper more intrinsically valuable in a professional point of view, and you will, therefore, exercise your editorial sagacity whether you insert it or reject this one, and although you may take "the will for the deed," if you decide the latter verdict, all I ask of your courtesy is to give this "Roman honors," or in other words, a fiery ordeal.

14 *Devonshire Place, Brighton, Nov.* 15, 1853.

